# Characteristics of the first metatarsophalangeal joint in gout and asymptomatic hyperuricaemia: a cross-sectional observational study

**DOI:** 10.1186/s13047-015-0091-8

**Published:** 2015-08-18

**Authors:** Sarah Stewart, Nicola Dalbeth, Alain C. Vandal, Keith Rome

**Affiliations:** Department of Podiatry, Health & Rehabilitation Research Institute, Auckland University of Technology, Private Bag 92006, Auckland, 1142 New Zealand; Faculty of Medical and Health Sciences, The University of Auckland, Private Bag 92019, Auckland, 1142 New Zealand; Department of Rheumatology, Auckland District Health Board, P.O. Box 92189, Auckland, New Zealand; Department of Biostatistics & Epidemiology, Faculty of Health and Environmental Sciences, Auckland University of Technology, Private Bag 92006, Auckland, 1142 New Zealand; Health Intelligence & Informatics, Ko Awatea, Counties Manukau Health, Private Bag 93311, Auckland, 1640 New Zealand

**Keywords:** Gout, First metatarsophalangeal joint, Patient-reported outcomes

## Abstract

**Background:**

This study aimed to identify patient-reported outcomes and clinician-assessed characteristics of the first metatarsophalangeal joint (1MTPJ) in people with gout and with asymptomatic hyperuricaemia by comparing them to normouricaemic controls.

**Methods:**

Twenty four people with gout (without current symptoms of acute arthritis), 29 with asymptomatic hyperuricaemia and 34 age- and sex-matched controls participated in this cross-sectional observational study. Patient-reported outcomes included 1MTPJ pain, foot pain and disability, body pain, lower limb function, activity limitation and overall wellbeing. Clinician-assessed characteristics of the 1MTPJ included range of motion (ROM), plantar- and dorsi-flexion force, foot posture, temperature and hallux valgus severity.

**Results:**

Compared to controls, participants with gout reported greater 1MTPJ pain (*p* = 0.014), greater foot pain and disability (*p* < 0.001), increased odds of having disabling foot pain (odds ratio (OR) 13.4, *p* < 0.001), decreased lower limb function for daily living (*p* = 0.002) and recreational (*p* < 0.001) activities, increased activity limitation (*p* = 0.002), reduced overall wellbeing (*p* = 0.034), reduced ROM (*p* < 0.001), reduced plantarflexion force (*p* = 0.012), increased 1MTPJ plantar (*p* = 0.004), dorsal (*p* = 0.003) and medial (*p* = 0.004) temperature and had increased odds of having more severe hallux valgus (OR 0.3 *p* = 0.041). Compared to controls, participants with asymptomatic hyperuricaemia had increased odds of having disabling foot pain (OR 4.2, *p* = 0.013), increased activity limitation (*p* = 0.033), decreased lower limb function for daily living (*p* = 0.026) and recreational (*p* = 0.010) activities, increased 1MTPJ plantarflexion force (*p* = 0.004) and a more pronated foot type (*p* = 0.036).

**Conclusions:**

People with gout demonstrate 1MTPJ-specific changes indicative of subclinical inflammation, even in the absence of acute arthritis. People with asymptomatic hyperuricaemia, who exhibit no features or symptoms of gout, also report high levels of foot- and lower limb-related pain and disability.

## Background

Gout is the most common form of inflammatory arthritis in middle-aged men [1, 2]. Abnormally high levels of urate in the blood, termed hyperuricaemia (defined as ≥0.41 mmol/L), is the most important risk factor in the development of gout [3]. The prevalence of gout and hyperuricaemia is increasing worldwide [4–7]. Hyperuricaemia can lead to the formation and deposition of monosodium urate (MSU) crystals in joints and soft tissues, and consequent clinical manifestations of gout, including episodes of acute gouty arthritis and tophus formation [8, 9]. Not all individuals with hyperuricaemia develop clinical features of gout [10–12]. However, recent imaging studies have reported the presence of MSU crystal deposition and subclinical joint- and extra-articular-damage in people with asymptomatic hyperuricaemia [13–19]. The clinical significance of these findings is currently unclear [20].

Acute gouty arthritis most commonly affects the first metatarsophalangeal joint (1MTPJ) [11, 21–23]. Advanced imaging studies have shown MSU crystal deposition frequently occurs within this joint not only in people with gout [24–26], but also in people with asymptomatic hyperuricaemia [14, 16]. People with gout report significant foot pain and report impairments and disability with everyday activities, including walking [27, 28]. In fact, they exhibit plantar pressure patterns and gait strategies consistent with an attempt to offload pain at the 1MTPJ [29]. Despite the importance of 1MTPJ function, particularly during the propulsive phase of gait [30, 31], the effect of gout and asymptomatic hyperuricaemia on patient-reported outcomes and clinical characteristics of the joint is unclear. This study therefore aims to identify patient-reported and clinician-assessed characteristics of the 1MTPJ in people with gout and people with asymptomatic hyperuricaemia by comparing them to normouricaemic controls.

## Methods

### Participants

This investigation was a cross-sectional observational study. Gout participants were recruited from Auckland District Health Board, Auckland, New Zealand. All participants fulfilled the 1977 preliminary American Rheumatism Association classification criteria for gout [21]. Participants without gout were recruited from Auckland University of Technology (AUT) staff. Non-gout participants underwent serum urate capillary testing on the day of the study using a Reflotron® Plus (Roche Diagnostics Ltd., New Zealand) and were stratified into either the asymptomatic hyperuricaemic group (serum urate ≥0.41 mmol/L) or the normouricaemic control group (serum urate <0.41 mmol/L). The three groups were age-and sex-matched. Participants were excluded if they were under 20 years of age; had a history of other inflammatory arthritis; were experiencing acute arthritis at the time of the clinical visit; had foot and/or ankle surgery in the previous 3 months; had a history of 1MTPJ surgery; lower limb amputation; or were unable to walk 10 m unaided. Ethical approval for the study was obtained from the AUT Ethics Committee (13/100) and locality assessment was obtained from Auckland District Health Board (A + 5891). All participants provided written informed consent prior to data collection.

All data were collected by a single researcher and registered podiatrist (SS). Demographic data were obtained from all participants including age, gender, ethnicity, body mass index (BMI), current medications and medical history. Additionally, gout disease characteristics were documented for gout participants including disease duration, flare history, and tophus presence.

### Patient-reported outcomes

Both right and left 1MTPJ pain, general body pain and patient global over the past week were assessed using 100 mm Visual Analogue Scales (VAS). Foot pain and disability was assessed using the 19-item Manchester Foot Pain and Disability Index (MFPDI) [32]. Each item was answered ‘none of the time’ (scored as 0), ‘on some days’ (scored as 1) or ‘on most/every day(s)’ (scored as 2) in the past month and a total score out of 38 was summated for each participant. Additionally, it was noted whether each participant had the presence of disabling foot pain, defined as at least one item scored as 1 or 2 [32]. The Health Assessment Questionnaire - Disability Index (HAQ-DI) [33] was used to measure activity limitation in which participants were asked to rate their ability to perform 10 tasks in the past week (without difficulty = 0, some difficulty = 1, much difficulty = 2, or unable = 3). The scores were summated and divided by 10 to give an overall value between 0 (minimal loss of function) and 3 (completely disabled). The Lower Limb Task Questionnaire (LLTQ) [34] was used to measure lower limb function related to two sections: activities of daily living (LLTQ daily) and recreational activities (LLTQ recreational). For each section participants were asked to rate their difficulty with 10 activities in the past 24 h (unable = 0, severe difficulty = 1, moderate difficulty = 2, mild difficulty = 3, and no difficulty = 4) from which a total score out of 40 was calculated.

### Clinician-assessed outcomes

Passive, non-weight-bearing 1MTPJ dorsiflexion range of motion (ROM) was measured using a hand-held goniometer (Whitehall Manufacturing Ltd., California, USA) in accordance with the procedure outlined by Hopson and McPoil [35]. Participants were positioned seated with knees extended and the ankle in neutral. Lines were drawn on the medial aspect of the foot along the sagittal bisections of the first metatarsal and proximal phalanx. The examiner applied a dorsiflexion force to the hallux until it could no longer be passively moved into further extension. The angle between the two bisection lines was measured from the goniometer. Three repeated measurements of right and left feet were taken.

Isometric muscle force for plantarflexion and dorsiflexion of the 1MTPJ was measured using a CITEC hand-held dynamometer (CIT Technics, Haren, Netherlands). Participants were positioned seated with knees extended and the foot stabilised in a custom-made device comprised of two wooden boards angled at 90°. The plantar foot was positioned against the vertical board with the ankle in a neutral position. Velcro straps were applied across the dorsum of the foot and lower leg, to isolate the 1MTPJ and to ensure the lower leg was held stationary. Strength was assessed using the ‘make’ technique in which the examiner held the dynamometer stationary while the participant exerted maximal force against it [36]. The dynamometer was positioned against the plantar aspect of the interphalangeal joint during plantarflexion testing and on the dorsal aspect of the hallux during dorsiflexion [37]. Three consecutive contractions of three to five seconds were recorded for each muscle group for each foot.

Hallux valgus severity was assessed using the Manchester Scale [38] which is comprised of four photographs graded as 0 being ‘no deformity’, 1 being ‘mild deformity’, 2 being ‘moderate deformity’ and 3 being ‘severe deformity’. The participant was asked to stand in a relaxed weight-bearing position while the examiner used the photographs to grade the deformity on each foot.

Foot type was assessed using the 6-item Foot Posture Index (FPI-6) [39] with the participant standing in a relaxed weight-bearing position. Each FPI criterion was scored on a five-point scale (−2 to +2) and a total score calculated for each foot ranging from −12 (highly supinated) to +12 (highly pronated).

Temperature of the 1MTPJ was measured using a DermaTemp 1001 (Exergen Corporation, Massachusetts), which is a hand-held infrared thermographic scanner with an in-built sensor. Participants were given adequate equilibration time in the room (thermostatically controlled at 22 °C ±2 °C). Temperatures were recorded from medial, dorsal and plantar sites of the 1MTPJ. Three readings for each site were repeated for each foot.

Where repeated measurements were taken, they were not averaged, but instead included as separate observations in the analysis as described below.

### Data analysis

Demographic and medical data were described as mean (SD) for continuous data and frequency (%) for categorical data. All continuous outcomes were reviewed for normality using the residuals from a linear model, which included relevant demographic covariates and the participant group as the independent variables. Appropriate regression models were identified for each outcome measure. Linear regression models were used for all continuous outcome measures. For the presence of disabling foot pain (a dichotomous outcome measure) logistic regression was used. For hallux valgus severity, an ordinal outcome measure, multinomial regression with cumulative logit link was used. Where appropriate, models accounted for repeated measures taken from right and left feet of each participant through using a mixed models approach in which a participant-specific random effect and participant-nested random effects for foot-side were added to the model. This analysis produces results identical to an analysis of measures averaged for each foot-side that would allow for a between-foot-side correlation, and also allows for any reweighting required due to missing values. For 1MTPJ temperature, which was measured at three sites (forming a natural vector of related variables) in addition to the participant and foot-side random effects, a heterogeneous compound symmetry covariance structure was employed, which allowed for separate variances for each site, as well as different covariances (but equal correlations) between each pair of sites.

Adjustments for gender, age group and ethnicity, which were entered into each model simultaneously, were considered only if their level of observed significance achieved at least 10 % on an F-test (or equivalent deviance test (i.e. Wald test) for categorical variables). Potential covariates were also explored by reviewing box plots of random effects by covariate group. A single-adjusted model was sought for each category of clinically-assessed outcome measures (i.e. patient-reported outcomes, structural and functional outcomes and neurovascular outcomes). Two contrasts were considered: gout vs. control and asymptomatic hyperuricaemia vs. control, which were always tested separately. All hypothesis tests (excluding covariate testing) were carried out at a 5 % level of significance against two-sided alternatives. No adjustment for multiplicity was used, but all test-statistics, their null distributions and their observed significance levels were reported. Data were analysed using IBM SPSS Statistics version 20 and SAS version 9.3.

## Results

A total of 87 participants were included with 24 in the gout group, 29 in the asymptomatic hyperuricaemic group and 34 controls. Demographic and clinical characteristics for the three groups are shown in Table 1. All participants were male with a mean (SD) age of 58 (15) years and predominantly of European ethnicity (*n* = 68, 81 %). The control group had a significantly lower mean BMI compared to the gout (*p* < 0.001) and asymptomatic hyperuricaemic participants (*p* < 0.001). Compared to controls, participants with gout had a significantly higher frequency of NSAID use (*p* = 0.004). The control group had a significantly lower prevalence of hypertension compared to the gout (*p* = 0.001) and asymptomatic hyperuricaemic groups (*p* = 0.023) and a significantly lower prevalence of cardiovascular disease compared to the gout group (*p* = 0.019). People with gout also had significantly higher mean tender (*p* = 0.032) and swollen joints counts (*p <* 0.001) compared to controls.

**Table 1:** Demographic and medical characteristics

Variable		Gout	Asymptomatic hyperuricaemia	Control
N		24	29	34
Gender, male, n (%)		24 (100 %)	29 (100 %)	34 (100 %)
Age, years, mean (SD)		58 (13)	58 (19)	58 (14)
Ethnicity, n (%)		European 14 (58 %)	European 24 (83 %)	European 30 (88 %)
		Maori 1 (4 %)	Maori 0 (0 %)	Maori 1 (3 %)
		Pacific 5 (21 %)	Pacific 3 (10 %)	Pacific 0 (0 %)
		Asian 4 (17 %)	Asian 2 (7 %)	Asian 3 (9 %)
BMI, kg/m^2^, mean (SD)		30.2 (4.0)*	29.3 (5.9)*	25.0 (2.9)
Diuretic use, n (%)		3 (12 %)	7 (24 %)	4 (12 %)
NSAID use, n (%)		14 (58 %)*	11 (38 %)	7 (21 %)
Prednisone use, n (%)		5 (21 %)	0 (0 %)	0 (0 %)
Hypertension, n (%)		17 (70 %)*	16 (55 %)*	9 (26 %)
Cardiovascular disease, n (%)		7 (29 %)*	5 (17 %)	1 (3 %)
Diabetes, n (%)		4 (17 %)	1 (3 %)	2 (6 %)
Urate, mmol/l	Mean (SD)	0.35 (0.10)	0.46 (0.05)*	0.32 (0.06)
	Range	0.24 - 0.63	0.41 - 0.63	0.20 - 0.40
1MTPJ tenderness, n (%)	Right	4 (17 %)	1 (0 %)	0 (0 %)
	Left	3 (12 %)	0 (0 %)	1 (3 %)
1MTPJ swelling, n (%)	Right	1 (4 %)	0 (0 %)	0 (0 %)
	Left	0 (0 %)	0 (0 %)	0 (0 %)
66/68 joint count, mean (SD)	Tender	2.7 (6.1)*	1.5 (1.9)	0.6 (1.2)
	Swollen	1.0 (1.7)*	0.2 (0.7)	0.0 (0.0)

*Significantly different from controls (*p* < 0.05)

Disease characteristics for the gout group are shown in Table 2. Gout participants were found to have a long disease duration with a mean (SD) of 17 (11) years, with 71 % (*n* = 17) having tophaceous gout and 96 % (*n* = 23) on urate lowering therapy. The majority of participants with gout reported a history of acute 1MTP arthritis on either foot (*n* = 21, 88 %).

**Table 2:** Gout disease characteristics

Variable	Gout
Classification criteria	Aspirate proven 6 (25 %)
	Clinical criteria 18 (75 %)
Disease duration, years, mean (SD)	17 (11)
Age of onset, years, mean (SD)	41 (18)
Acute flares in preceding 3 months, mean (SD)	1.3 (1.4)
1MTPJ flares in preceding 3 months, n (%)	6 (25 %)
History of 1MTPJ flares, n (%)	21 (88 %)
Presence of subcutaneous tophi, n (%)	17 (71 %)
Presence of 1MTPJ tophi, n (%)	6 (25 %)
Number of tophi in feet, mean (SD)	1.9 (3.5)
Total number of tophi, mean (SD)	6.1 (8.7)
Colchicine use, n (%)	13 (54 %)
Urate lowering therapy^a^, n (%)	23 (96 %)
Allopurinol use, n (%)	19 (79 %)
Probenecid use, n (%)	3 (12 %)
Benzbromarone use, n (%)	2 (8 %)
Febuxostat use, n (%)	2 (8 %)

^a^3 patients were taking >1 urate lowering agent

The distribution of residuals from the linear models for all outcome measures demonstrated sufficient normality to carry out parametric testing. All final models were adjusted for age group. Table 3 displays the mean estimates and inferential statistics for all patient-reported outcomes. Compared to controls, participants with gout reported significantly greater 1MTPJ pain (*p* = 0.014), greater patient global scores (*p* = 0.034), a greater HAQ-DI score (*p* = 0.002), a greater LLTQ daily score (*p* = 0.002), a greater LLTQ recreational score (*p* < 0.001), a greater MFPDI score (*p* < 0.001), and a higher odds of having disabling foot pain (OR 13.4; *p* < 0.001). Participants with asymptomatic hyperuricaemia also reported a significantly greater HAQ-DI score (*p* = 0.033), a greater LLTQ daily score (*p* = 0.026), a greater LLTQ recreational score (*p* = 0.010) and had a higher odds of having disabling foot pain (OR 4.2 *p* = 0.013), compared to controls.

**Table 3:** Patient-reported outcomes

Parameter		Mean estimate	Diff.	95 % CI		*p*
				Lower	Upper	
1MTP pain VAS (mm)	Control (ref.)	1.7				
	Gout	8.4	6.7	1.4	12.0	0.014
	Asymptomatic hyperuricaemia	6.6	4.9	−0.1	10.0	0.055
General pain VAS (mm)	Control (ref.)	18.0				
	Gout	21.8	3.8	−9.9	17.5	0.581
	Asymptomatic hyperuricaemia	29.2	11.3	−1.7	24.3	0.088
Patient global VAS (mm)	Control (ref.)	11.5				
	Gout	23.6	12.0	0.9	23.1	0.034
	Asymptomatic hyperuricaemia	21.3	9.8	−0.8	20.3	0.068
HAQ-DI	Control (ref.)	0.11				
	Gout	0.44	0.33	0.13	0.54	0.002
	Asymptomatic hyperuricaemia	0.32	0.21	0.02	0.41	0.033
LLTQ - daily	Control (ref.)	38.6				
	Gout	33.3	−5.3	−8.6	−2.0	0.002
	Asymptomatic hyperuricaemia	35.0	−3.6	−6.8	−0.4	0.026
LLTQ – recreational	Control (ref.)	34.2				
	Gout	20.8	−13.4	−19.0	−7.9	<0.001
	Asymptomatic hyperuricaemia	27.2	−7.0	−12.3	−1.8	0.010
MFPDI	Control (ref.)	1.826				
	Gout	13.3	11.5	7.7	15.3	<0.001
	Asymptomatic hyperuricaemia	3.0	1.2	−2.4	4.8	0.511
			Odds ratio	95 % CI for OR		*p*
				Lower	Upper	
Presence of disabling foot pain	Control (ref.)					
	Gout		13.4	3.69	48.68	<0.001
	Asymptomatic hyperuricaemia		4.2	1.36	12.8	0.013

Table 4 displays the mean estimates and inferential statistics for all clinician-assessed outcomes. Compared to controls, participants with gout had significantly reduced 1MTPJ ROM (*p* < 0.001), reduced 1MTPJ plantarflexion force (*p* = 0.012), an increased odds of having more severe hallux valgus (OR 0.3; *p* = 0.041) and increased temperature at the plantar (*p* = 0.004), dorsal (*p* = 0.003), and medial (*p* = 0.004) aspects of the 1MTPJ. Participants with asymptomatic hyperuricaemia had significantly greater 1MTPJ plantarflexion force (*p* = 0.004) and a higher FPI score (*p* = 0.036), compared to controls.

**Table 4:** Clinician-assessed outcomes

Parameter		Mean estimate	Diff.	95 % CI		*p*
				Lower	Upper	
ROM (°)	Control (ref.)	77.6				
	Gout	59.7	−17.9	−26.8	−8.9	<0.001
	Asymptomatic hyperuricaemia	76.8	−0.8	−9.3	7.7	0.853
Plantarflexion force (N)	Control (ref.)	92.0				
	Gout	71.3	−20.7	−36.9	−4.6	0.012
	Asymptomatic hyperuricaemia	114.8	22.8	7.5	38.1	0.004
Dorsiflexion force (N)	Control (ref.)	57.3				
	Gout	58.0	0.8	−10.7	12.2	0.896
	Asymptomatic hyperuricaemia	65.4	8.1	−2.7	19.0	0.139
Foot Posture Index	Control (ref.)	+4.8				
	Gout	+6.2	1.3	−0.4	3.1	0.134
	Asymptomatic hyperuricaemia	+6.6	1.8	0.1	3.4	0.036
Plantar temperature (°C)	Control (ref.)	24.3				
	Gout	26.2	1.9	0.6	3.1	0.004
	Asymptomatic hyperuricaemia	25.1	0.8	−0.5	2.0	0.218
Dorsal temperature (°C)	Control (ref.)	25.8				
	Gout	27.7	1.9	0.6	3.1	0.003
	Asymptomatic hyperuricaemia	26.5	0.6	−0.6	1.9	0.295
Medial temperature (°C)	Control (ref.)	25.2				
	Gout	27.0	1.8	0.6	3.1	0.004
	Asymptomatic hyperuricaemia	25.9	0.8	−0.4	2.0	0.219
				95 % CI for OR		*p*
				Lower	Upper	
Hallux Valgus Severity^a^	Control (ref.)					
	Gout		0.284	0.085	0.947	0.041
	Asymptomatic hyperuricaemia	0.968	0.968	0.296	0.957	

^a^Reference category: none (i.e. grade 0). The odds ratio represents the odds of the diagnostic group moving up one category of severity, compared to the control group moving up one category of severity

## Discussion

This study investigated patient-reported outcomes and clinician-assessed characteristics of the 1MTPJ in people with gout and people with asymptomatic hyperuricaemia. Despite the absence of current symptoms of acute arthritis in the gout participants and an absence of any signs or symptoms of gout in the asymptomatic hyperuricaemic participants, both groups reported high levels of foot- and lower limb-related pain and disability. Additionally, people with gout demonstrated 1MTPJ-specific changes related to pain, joint motion, muscle strength, hallux valgus severity and temperature.

Clinical symptoms in gout are generally associated with acute episodes of painful inflammatory arthritis, most often at the 1MTPJ [21], while intercritical periods are considered to be ‘asymptomatic’ remissive phases [40, 41]. However, our findings, which support existing research [42, 43], suggest that 1MTPJ pain may be a chronic and persistent foot problem in people with gout. These results may be explained by the presence of subclinical inflammation, which is further emphasised by the increased 1MTPJ temperature observed in the gout participants in this study [44]. It has been well established that MSU crystals, which promote the inflammatory response evident in acute gout, are also present in synovial fluid during intercritical periods [45]. Furthermore, imaging studies have frequently observed synovitis in gout patients in the absence of clinically evident inflammation [25, 46, 47]. The clinical relevance of persistent inflammation at the 1MTPJ in people with gout is uncertain.

This study has also identified a number of structural and functional changes at the 1MTPJ in people with gout. Although restricted 1MTPJ motion may be a result of surrounding synovial inflammation or a pain-avoidance mechanism, previous research has regarded reduced 1MTPJ motion in people with gout as a clinical measure of osteoarthritis [43]. It has been suggested that osteoarthritis may predispose to localised MSU crystal deposition and thus may explain the tendency for gout to affect the 1MTPJ [43, 44]. However, it remains uncertain whether osteoarthritis precedes gout or whether joint damage results from chronic gouty arthritis and/or mechanical obstruction by tophi [45].

Participants with gout also exhibited a reduction in 1MTPJ plantarflexion force. Considering the importance of 1MTPJ plantarflexion force during the forward transfer of body weight in normal walking [46], we speculate that reduced strength in this muscle group may be related to the apropulsive gait patterns previously observed in people with gout who demonstrated reduced peak pressure beneath the hallux [26]. The authors proposed this was a pain-avoidance strategy, which would reduce plantarflexor muscle activity and may lead to disuse muscle atrophy.

Although participants with asymptomatic hyperuricaemia did not display the 1MTPJ-specific changes observed in the gout group, they did report greater overall foot pain and disability, reduced lower limb function and increased activity limitation compared to the normouricaemic controls. It is unclear whether this is a direct result of chronically elevated serum urate and subclinical MSU deposition, inflammation and tissue damage [13–18] or related to co-existing conditions including hypertension, obesity, cardiovascular disease and diabetes, which have a marked association with hyperuricaemia and may display clinical manifestations in the foot and lower limb [48–54].

The association between chronically elevated serum urate levels and patient-reported outcomes is unclear and currently there is no consensus on the treatment of asymptomatic hyperuricaemia due to the small number of hyperuricaemic individuals that develop gout [10, 55] and the side effects of treatment with urate lowering therapy [56, 57]. However, the low-grade systemic inflammation, which has been reported in patients with asymptomatic hyperuricaemia [58, 59] along with the results of the current study highlight the need for further research in this area, particularly in the evaluation of treatment strategies aimed at improving patient-reported outcomes.

Our findings should be considered in light of several limitations. Firstly, our study included only male participants so our results cannot be generalisable to both genders. Secondly, we did not exclude participants with diabetes, cardiovascular disease and hypertension, which may have impacted our results. The majority of patients with gout had advanced disease with tophi, and it is possible that less severe 1MTPJ disease may be present in those with early gout or without gouty tophi. Lastly, the cross-sectional nature of our study design limits the ability to determine the cause and effect relationship between 1MTPJ characteristics and different disease states.

Further research may employ methods of advanced imaging to identify subclinical characteristics of gouty arthritis at the 1MTPJ in people with gout and people with asymptomatic hyperuricaemia in correlation to clinically-assessed features of the joint. Considering the lower-limb related functional impairments reported by gout and asymptomatic hyperuricaemic participants in the current study, future research may also investigate how this is reflected in gait parameters. The findings from this study may be useful in directing future research, which evaluates the efficacy of non-pharmacological treatment strategies, such as footwear [60, 61] and orthoses, which specifically target the 1MTPJ in combination with urate lowering therapy.

## Conclusion

In conclusion, this study has shown that 1MTPJ pain is commonly reported by people with gout during intercritical periods. Clinician-assessed characteristics of the joint, including reduced motion and increased temperature, are indicative of subclinical inflammation and highlight the impact of gout on the structure and function of the 1MTPJ. This study has also shown that people with asymptomatic hyperuricaemia, who do not display any signs or symptoms of gout, also experience considerable foot- and lower limb-related pain and impairment and report greater activity limitation when compared to normouricaemic controls.
